# Overnight stay in the emergency department and mortality in sepsis patients

**DOI:** 10.7717/peerj.21880

**Published:** 2026-11-10

**Authors:** Junnan Xu, Yilong Shi, Xinyuan Yu, Fengyu Chen, Liang Wang, Chan Chen, Zhiyi Wang, Jie Weng, Yingbin Deng

**Affiliations:** 1 Department of Emergency Medicine, The Second Affiliated Hospital and Yuying Children’s Hospital of Wenzhou Medical University, Wenzhou, China; 2 Department of General Practice, Lu’an Hospital of Anhui Medical University, Lu’an, China; 3 Department of Public Health, Marshall University, West Virginia, United States; 4 South Zhejiang Institute of Radiation Medicine and Nuclear Technology, Wenzhou, China; 5 Geriatric Medicine, The First Affiliated Hospital of Wenzhou Medical University, Wenzhou, China; 6 Wenzhou Key Laboratory of Precision General Practice and Health Management, Wenzhou, China; 7 Department of General Practice, Beijing Tsinghua Changgung Hospital, School of Clinical Medicine, Tsinghua University, Beijing, China; 8 Department of General Practice, The Second Affiliated Hospital and Yuying Children’s Hospital of Wenzhou Medical University, Wenzhou, China

**Keywords:** Overnight stay in the emergency department, Sepsis, Mortality

## Abstract

**Background:**

Sepsis management in the emergency department (ED) faces challenges due to overcrowding and prolonged boarding, yet the impact of overnight ED stays on outcomes remains unclear. This study evaluated whether sepsis patients who spend a night in the ED waiting for admission to a ward/intensive care unit (ICU) are at increased risk of in-hospital mortality or morbidity.

**Methods:**

This was a retrospective cohort study of sepsis patients who visited the ED and were admitted to the hospital between January 2021 and August 2024 across the ED of a large general hospital in China. Patients were stratified into an overnight ED stay (midnight–8:00 AM) and a non-overnight (admitted to a ward/ICUs before midnight) group. The primary outcome was in-hospital mortality; secondary outcomes included septic shock, ICU admission, invasive mechanical ventilation (IMV) and length of stay (LOS). Multivariable logistic regression and sensitivity analyses were used to compare end points between groups.

**Results:**

A total of 1,208 sepsis patients were enrolled in the study, including 503 (42%) female and 705 (58%) male, with 624 (51.7%) in the overnight ED stay group and 584 (48.3%) in the non-overnight ED stay group. Baseline characteristics were balanced between groups. Unadjusted in-hospital mortality was similar (21.2% *vs*. 21.7%; risk difference (RD) 0.59 [−4.04 to 5.23]). Adjusted logistic regression analyses confirmed no mortality association (adjusted odds ratio (aOR) (95% confidence interval (CI)), 1.090 [0.805–1.477]; *P* = 0.579). No higher risk was observed for secondary outcomes: septic shock (aOR (95% CI), 1.020 [0.747–1.393]; *P* = 0.903), ICU admission (aOR 1.362 [0.983–1.895]; *P* = 0.064), or IMV (aOR (95% CI), 1.048 [0.710–1.549]; *P* = 0.813). Compliance with the Surviving Sepsis Campaign (SSC) guidelines was comparable between groups(31.6% *vs*. 29.1%; RD, −2.46 [−7.63 to 2.73]). These findings were consistent across subgroups and sensitivity analyses.

**Conclusions:**

In this retrospective cohort study, overnight ED boarding was not associated with increased in-hospital mortality or morbidity among sepsis patients when care adhered to SSC guidelines. These results suggest that high compliance with the SSC 1-h bundle may help mitigate potential risks associated with overnight ED boarding.

## Introduction

Sepsis remains a global healthcare priority, accounting for substantial morbidity and mortality burdens across healthcare systems worldwide (Evans et al., 2021; Jouffroy, Vivien & Gueye, 2024). The critical importance of early clinical recognition and prompt implementation of time-sensitive interventions, such as antimicrobial therapy and hemodynamic optimization, has been well established as fundamental to improving patient outcomes (Jouffroy, Vivien & Gueye, 2024; Weng et al., 2024; Zhou et al., 2024; Pak et al., 2024). The emergency department (ED) serveas as the frontline for sepsis management, with initial diagnostic and therapeutic decisions significantly influencing clinical trajectories (Gaieski et al., 2017). However, evolving operational challenges in emergency care environments, particularly increasing rates of ED overcrowding and extended treatment delays, now threaten to compromise the delivery of these time-critical interventions (Peltan et al., 2019; Shin et al., 2013).

Extended ED boarding durations, particularly overnight hospitalization in acute care settings, have emerged as independent predictors of multiple adverse clinical outcomes across diverse patient cohorts (Mohr et al., 2020; Singer et al., 2011). In a recent multicenter cohort study, overnight stay in the ED was independently associated with increased in-hospital mortality and morbidity, highlighting the importance of minimizing overnight stays in elderly patients (Roussel et al., 2023). Patients with sepsis are particularly susceptible to the consequences of extended ED boarding, including disruptions in timely antibiotic administration, fluid resuscitation, and other guideline-recommended interventions (Weng et al., 2024; Blank et al., 2024). Additionally, the environmental stressors of the ED—such as noise, light exposure, limited nursing resources and prolonged immobilization on a trolley—may exacerbate the risks of organ dysfunction and secondary complications during critical early stages of sepsis management (Mannion, Molloy & O’Caoimh, 2019; Prendiville et al., 2020; Zhou et al., 2023). Importantly, compliance with sepsis bundles was significantly associated with patient outcomes, while bundle compliance was inconsistent in different ED time periods (You et al., 2022; Ranzani et al., 2020; Almeida et al., 2013). Despite these findings, the mortality and morbidity of sepsis patients who spend the night in the ED while waiting for admission are unknown.

The aim of this study was to evaluate the differences in in-hospital mortality and morbidity between sepsis patients who spent their first hospitalized night in the ED and those who spent it in a hospital ward or intensive care unit (ICU). Additionally, we planned subgroup analyses to explore effect modification by factors such as sex, age, and disease severity, based on prior hypotheses regarding potential heterogeneity in the association between ED boarding and outcomes.

## Methods

### Study design and setting

This retrospective cohort study was conducted at the Second Affiliated Hospital and Yuying Children’s Hospital of Wenzhou Medical University, a large comprehensive tertiary hospital, and included sepsis patients admitted between January 2021 and August 2024. The study adhered to the guidelines for cohort studies to enhance reporting of observational epidemiological studies (STROBE) (von Elm et al., 2007). The study protocol was approved by the Ethics Review Committee of the Second Affiliated Hospital and Yuying Children’s Hospital of Wenzhou Medical University, and no informed consent was required due to the retrospective nature of the study (IRB# 2021-K-271-01).

### Study population

The study included adult patients (≥18 years) who presented to the ED with a diagnosis of sepsis, defined according to Sepsis-3 criteria as suspected infection accompanied by an increase in the Sequential Organ Failure Assessment (SOFA) score by ≥2 points from baseline (Rhodes et al., 2017). Eligibility was assessed by two trained clinicians using electronic health record data. Patients were eligible if they were subsequently admitted to a ward/ICU or remained in the ED overnight (midnight to 8:00 AM). Patients discharged home directly from the ED, transferred to another facility or nursing home, or admitted to a ward between midnight and 8:00 AM were excluded.

Patients who remained in the ED overnight (midnight to 8:00 AM) while awaiting transfer to a ward/ICU were classified into the overnight stay in the ED group, those transferred to ward/ICU before midnight were classified into the non-overnight stay in the ED group. This dichotomous definition was chosen to identify patients exposed to the sustained overnight period in the ED. We acknowledge that it does not distinguish variation in boarding duration within that window. To address this, the total ED length of stay (LOS) and the overnight-specific ED LOS were also analyzed as continuous variables to examine the association of boarding duration with outcomes.

### Outcomes

The primary outcome was in-hospital mortality, defined as death occurring during the hospitalization. Secondary outcomes included septic shock during the hospitalization, ICU admission, invasive mechanical ventilation (IMV), as well as ICU LOS, hospital LOS.

### Data collection

Electronic health records (EHRs) were reviewed to extract routinely collected data on patients with sepsis identified during the inclusion period. Data were extracted by trained clinicians using a standardized case report form with detailed inclusion/exclusion criteria and variable definitions. The case report form was pilot-tested on a small sample before full data collection. All extractors received standardized training, and regular team meetings were held to resolve any ambiguities or discrepancies. Although a formal inter-rater reliability assessment (*e.g*., kappa statistic) was not performed, the majority of collected variables were objective data points (vital signs, laboratory values, and timestamps) derived directly from the electronic health record, which minimized subjective interpretation.

Demographic information, Age-adjusted Charlson Comorbidity Index (aCCI), site of infection, and clinical parameters at ED presentation were collected. Vital signs recorded at presentation included mean arterial pressure (MAP), heart rate, respiratory rate, and body temperature. Key laboratory values were documented where available. The severity of illness was assessed using the Acute Physiology and Chronic Health Evaluation II (APACHE II) score and SOFA score.

Compliance with the Surviving Sepsis Campaign (SSC) 1-h bundle was defined as the completion of all applicable core components within 1 h of sepsis recognition: (1) measurement of serum lactate level, (2) drawing of blood cultures before antibiotic administration, (3) administration of broad-spectrum antibiotics, and (4) fluid resuscitation with at least 30 mL/kg crystalloid for patients with hypotension or elevated lactate, along with immediate initiation of vasopressors if mean arterial pressure targets were not met. Overall bundle compliance required all applicable components to be completed within the 1-h window.

Baseline characteristics and exposures were defined *a priori* and recorded in alignment with the study objectives. ED length of stay was defined as the time from ED arrival to departure for a hospital ward or other location. In-hospital length of stay was calculated from the time of ED arrival and included the time spent in the ED. In-hospital mortality data were retrieved from EHRs or hospital administrative databases as needed.

### Statistical analysis

Patient characteristics were summarized for the entire cohort and stratified by group (non-overnight *vs*. overnight stay in the ED). Continuous variables were presented as either mean with standard deviation (SD) or median with interquartile range (IQR), depending on the data distribution. Categorical variables were expressed as frequencies and percentages. Differences in patient characteristics between groups, along with 95% CIs, were calculated using the Agresti-Caffo method (Agresti & Caffo, 2000) for categorical variables and either the normal approximation or the Brookmeyer-Crowley method (Brookmeyer & Crowley, 1982) for continuous variables based on distributions.

The primary outcome was in-hospital mortality, while secondary outcomes included septic shock during the hospitalization, ICU admission, IMV, ICU LOS and hospital LOS. Differences in proportions between groups with corresponding 95% CIs were calculated using the Agresti-Caffo method (Agresti & Caffo, 2000).

To adjust for confounding variables, multivariable logistic regression was employed. Adjustment was performed for age, sex, Body Mass Index (BMI), aCCI, APACHE II score and SOFA score, site of infection, compliance with 1-h bundle. The findings are presented as adjusted odds ratio (aOR). The exposure variables of interest included the dichotomous overnight stay in the ED, as well as ED LOS and ED Overnight LOS, both treated as continuous variables, to assess the relationship between boarding duration and outcomes. Multiple linear regression analysis was performed to analyze the effect of overnight stay in the ED, ED LOS, ED overnight LOS on ICU LOS and hospital LOS. Additionally, corresponding multivariable models were constructed for all secondary outcomes and exposure variables. Given the multiple comparisons involving secondary outcomes and subgroup analyses, findings should be interpreted as exploratory, and no adjustments for Type I error inflation were made, as the analyses were hypothesis-generating rather than confirmatory.

To address potential confounding by indication, we performed sensitivity analyses using inverse probability of treatment weighting (IPTW) (Austin, 2011; Robins, Hernán & Brumback, 2000). A preplanned exploratory analysis was performed to evaluate whether patient characteristics, emergency interventions, or illness severity mediated the association between overnight stay in the ED and adverse outcomes in sepsis patients.

Multiple imputation was used for data filling. The number of missing values for all variables was less than 5%. All statistical tests were two-tailed, and *P* values < 0.05 were considered statistically significant. Statistical analyses were performed using R software (version 4.4.1).

## Results

The study initially screened 1,424 patients with sepsis and excluded 216 of them based on predetermined criteria, resulting in 1,208 patients. Of these, 624 (51.7%) stayed overnight in the ED and 584 (48.3%) were admitted to an inpatient ward before midnight (Fig. 1). The baseline characteristics of the overnight and non-overnight groups were relatively well balanced in terms of age (mean difference (MD), −1 [−3 to 1]), sex (risk difference (RD), −2.93 [−8.47 to 2.64]), body mass index (MD, −0.03 [−0.39 to 0.32]), vital signs (MAP: MD, 0 [0 to 1]; heart rate: MD, 0 [−3 to 3]; respiratory rate: MD, 0 [0 to 0]; body temperature: MD, 0.0 [−0.1 to 0.0]), or disease severity scores (APACHE II: MD, 0 [−1 to 0]; SOFA: MD, 0 [0 to 0]) (Table 1). However, albumin levels (MD, 0.9 [0.2 to 1.6]), creatinine (MD, −4.8 [−9.5 to −0.1]), and urea nitrogen (MD, −0.5 [−1.0 to 0.0]) were lower in the overnight group compared with the non-overnight group (Table 1).

**Figure 1:**
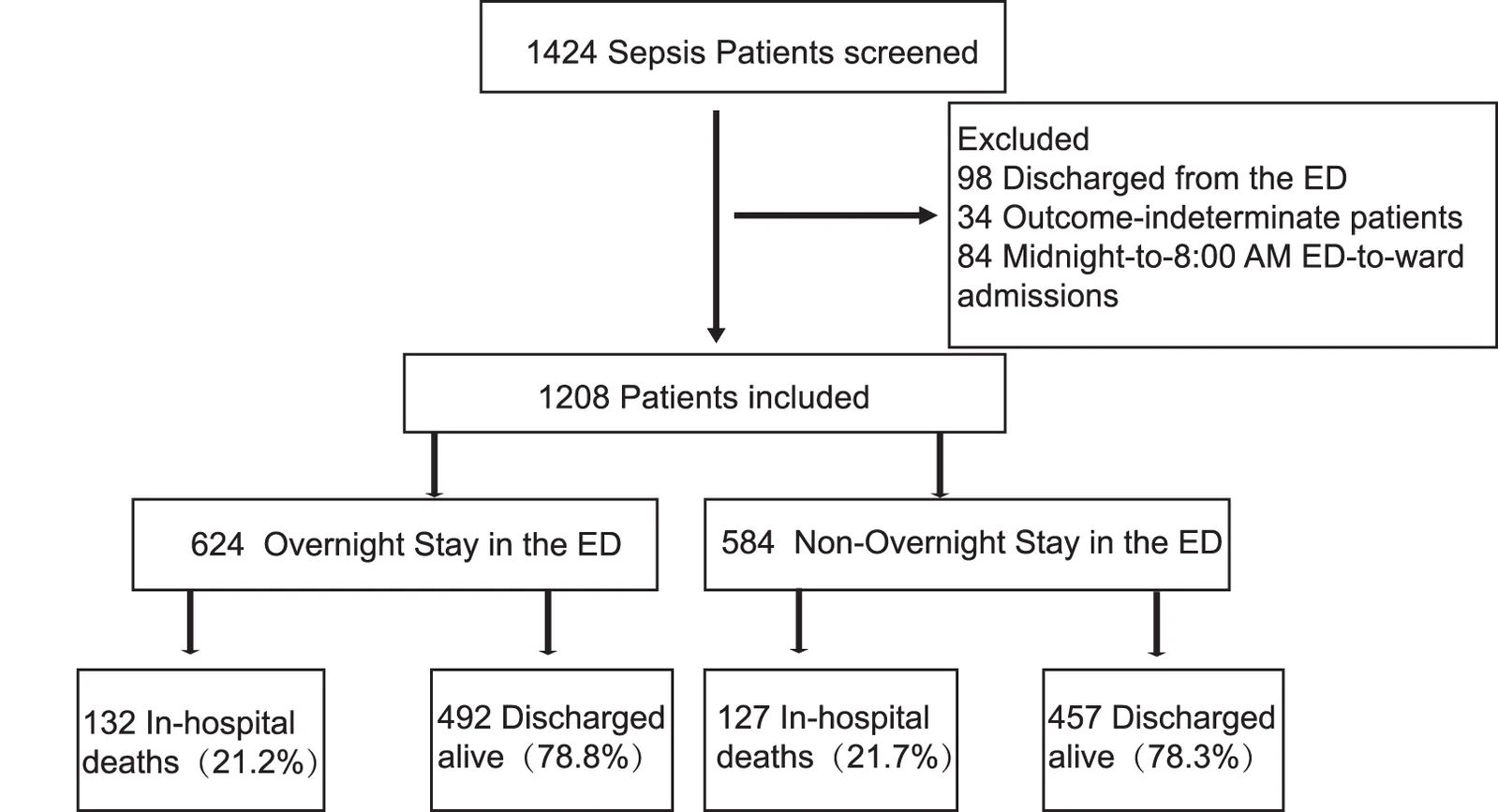
Subject inclusion/exclusion flow diagram.

**Table 1:** Characteristics of sepsis patients according to with *vs*. without overnight stay in the emergency department.

Characteristic	Non-overnight stay in the ED (*n* = 584)	Overnight stay in the ED (*n* = 624)	Difference (95% CI)
**Demographics**			
Age, (IQR, years)	66 [54; 76]	65 [53; 76]	−1 [−3 to 1]
Male sex, *n* (%)	332 (56.8)	373 (59.8)	−2.93 [−8.47 to 2.64]
BMI (IQR, kg/m^2^)	22.2 [20.4; 24.7]	22.2 [20.2; 24.6]	−0.03 [−0.39 to 0.32]
Missing data (%)	22 (3.8)	40 (6.4)	
**Vital signs**			
Body temperature, Median (IQR, °C)	36.9 [36.7; 37.2]	36.9 [36.7; 37.2]	0.0 [−0.1 to 0.0]
Missing data (%)	11 (1.9)	15 (2.4)	
MMAP, Median (IQR, mmHg)	89 [82; 94]	89.0 [82; 95]	0 [0–1]
Missing data (%)	3 (0.5)	2 (0.3)	
Heart rate, Median (IQR, bpm)	94 [78; 111]	94.0 [79; 110]	0 [−3 to 3]
Missing data (%)	2 (0.3)	5 (0.8)	
Respiratory rate, Median (IQR, rpm)	19[18; 20]	19.0 [18; 20]	0 [0–0]
Missing data (%)	14 (2.4)	13 (2.1)	
**Site of infection**			−3.56 [−8.52 to 1.41]
Pulmonary infection, *n* (%)	164 (28.1)	153 (24.5)	
Soft tissue infection, *n* (%)	23 (3.94)	27 (4.33)	
Urinary system infection, *n* (%)	76 (13.0)	87 (13.9)	
Intraperitoneal infection, *n* (%)	134 (22.9)	141 (22.6)	
Unknow, *n* (%)	150 (25.7)	174 (27.9)	
Other, *n* (%)	37 (6.34)	42 (6.73)	
**Laboratory tests**			
CRP, Median (IQR, mg/l)	86.9 [27.5; 164.0]	91.2 [29.8; 164.0]	1.68 [−5.75 to 9.81]
WBC, Median (IQR, *10^9^/l)	8.97 [5.74; 12.9]	8.62 [5.71; 13.4]	0.03 [−0.59 to 0.66]
PLT, Median (IQR, *10^12^/l)	3.76 [3.15; 4.24]	3.78 [3.30; 4.32]	0.06 [−0.03 to 0.15]
Hb, Median (IQR, g/l)	114.0 [93.0; 128.0]	114.0 [97.0; 130.0]	1.0 [−1.0 to 4.0]
ALT, Median (IQR, U/L)	27.0 [16.0; 56.0]	26.0 [15.0; 49.2]	−2 [−4 to 1]
AST, Median (IQR, U/L)	34.0 [20.0; 65.2]	30.0 [19.0; 60.0]	−2 [−5 to 0]
Alb, Median (IQR, g/L)	32.9 [28.5; 37.3]	33.8 [29.9; 37.5]	0.9 [0.2–1.6]
TBil, Median (IQR, μmol/L)	13.3 [8.40; 22.4]	12.3 [8.20; 22.1]	−0.5 [−1.5 to 0.4]
Cr, Median (IQR, μmol/L)	82.0 [59.5; 140]	76.9 [57.7; 125]	−4.8 [−9.5 to −0.1]
BUN, Median (IQR, mmol/L)	7.50 [4.85; 12.4]	7.00 [4.70; 10.8]	−0.5 [−1 to 0]
GLU, Median (IQR, mmol/L)	6.04 [4.87; 7.98]	5.86 [4.91; 7.76]	−0.01 [−0.24 to 0.21]
PCT, Median (IQR, ng/mL)	0.41 [0.00; 4.49]	0.41 [0.00; 4.09]	0 [0–0]
**Severity score**			
aCCI, Median (IQR)	4 [2; 5]	3 [1; 5]	0 [0–0]
APACHE II score, Median (IQR)	10 [8; 13]	10 [7; 13]	0 [−1 to 0]
SOFA score, Median (IQR)	3 [2, 4]	3 [2, 3]	0 [0–0]
**Outcome**			
Mortality, *n* (%)	127 (21.7)	132 (21.2)	0.59 [−4.04 to 5.23]
Shock, *n* (%)	104 (17.8)	106 (17.0)	0.82 [−3.46 to 5.11]
ICU admission, *n* (%)	177 (30.3)	191 (30.6)	−0.3 [−5.48 to 4.89]
Invasive mechanical ventilation, *n* (%)	100 (17.1)	97 (15.5)	0 [0–0]
*Length of ICU stay, Median (IQR, day)	5.18 [2.56; 15.8]	5.59 [2.34; 12.6]	−0.11 [−1.16 to 0.95]
Length of hospital stay, Median (IQR, day)	11.0 [6.17; 18.9]	10.7 [6.75; 18.9]	−0.03 [−0.97 to 0.91]

**Notes:**

* The computational analysis was exclusively performed on the intensive care unit (ICU)-admitted patient cohort.

CRP, C-reactive protein; WBC, white cell count; PLT, platelet, Hb, hemoglobin; ALT, alanine aminotransferase; AST, aspartate aminotransferase; Alb, albumin; TBil, total bilirubin; Cr, creatinine; BUN, blood urea nitrogen; GLU, blood glucose; PCT, procalcitonin.

### Primary and secondary outcomes

Unadjusted in-hospital mortality rates were similar between the overnight stay and non-overnight stay groups (21.2% *vs*. 21.7%; RD, 0.59 [−4.04 to 5.23]). After adjustment for age, sex, BMI, aCCI, APACHE II score, site of infection, and compliance with the 1-h sepsis bundle, no significant association was observed between overnight ED stay and mortality (aOR (95%CI), 1.090 [0.805–1.477]; *P* = 0.579) (Table 2). Similarly, overnight ED stay was not associated with increased risks of shock (aOR (95%CI), 1.020 [0.747–1.393]; *P* = 0.903), IMV (aOR (95% CI), 1.048 [0.710–1.549]; *P* = 0.813), or prolonged ICU LOS (β Coefficient (95% CI), −4.347 (−15.056 to 6.362); *P* = 0.425) or hospital LOS (β Coefficient (95% CI), −0.640 [−4.228 to 2.948]; *P* = 0.726) (Table 2). For ICU admission, the borderline *P*-value (*P* = 0.064) and effect size (aOR 1.362) suggest a potential clinically relevant trend that may warrant further investigation with larger samples. Furthermore, when analyzed as continuous variables, neither the ED LOS nor ED overnight LOS was significantly associated with an increased risk of any primary or secondary adverse outcome in the adjusted models (Table 2).

**Table 2:** Adjusted association of overnight stay in the ED, ED LOS, ED overnight LOS and adverse outcome patients with sepsis.

Variable	Mortality		Shock		ICU admission		IMV		*ICU LOS		Hospital LOS	
	Adjusted OR (95% CI)	*P* value	Adjusted OR (95%CI)	*P* value	Adjusted OR (95% CI)	*P* value	Adjusted OR (95%CI)	*P* value	β coefficient (95% CI)	*P* value	β coefficient (95% CI)	*P* value
Overnight stay in the ED	1.090 [0.805–1.477]	0.579	1.020 [0.747–1.393]	0.903	1.362 [0.983–1.895]	0.064	1.048 [0.710–1.549]	0.813	−4.347 [−15.056 to 6.362]	0.425	−0.640 [−4.228 to 2.948]	0.726
ED LOS	1.005 [0.992–1.018]	0.410	0.998 [0.984–1.011]	0.781	1.010 [0.996–1.023]	0.158	1.005 [0.987–1.022]	0.601	−0.067 [−0.527 to 0.394]	0.776	−0.028 [−0.181 to 0.124]	0.715
ED overnight LOS	1.011 [0.980–1.042]	0.490	0.993 [0.961–1.025]	0.656	1.015 [0.983–1.048]	0.363	0.999 [0.959–1.040]	0.981	−0.287 [−1.377 to 0.802]	0.605	−0.104 [−0.468 to 0.261]	0.577

**Notes:**

ED, Emergency department; LOS, length of stay; ICU, intensive care unit; IMV, invasive mechanical ventilation.

* This study was confined to the analysis of patients admitted to the ICU.

### Sensitivity analyses

To address potential covariances between SOFA and APACHE II scores, adjusting the secondary model for SOFA but not APACHE II produced consistent results (Table S1). Propensity score weighting (estimated using IPTW) achieved balanced baseline covariates (standardized mean difference <0.1; Fig. S1), and sensitivity analyses using alternative statistical methods confirmed the robustness of these findings (Tables S2 and S3). Subgroup analyses further demonstrated that sex, age, body mass index, comorbidities, disease severity, or CRP did not influence the results (Fig. 2, Figs. S2 and S3).

**Figure 2:**
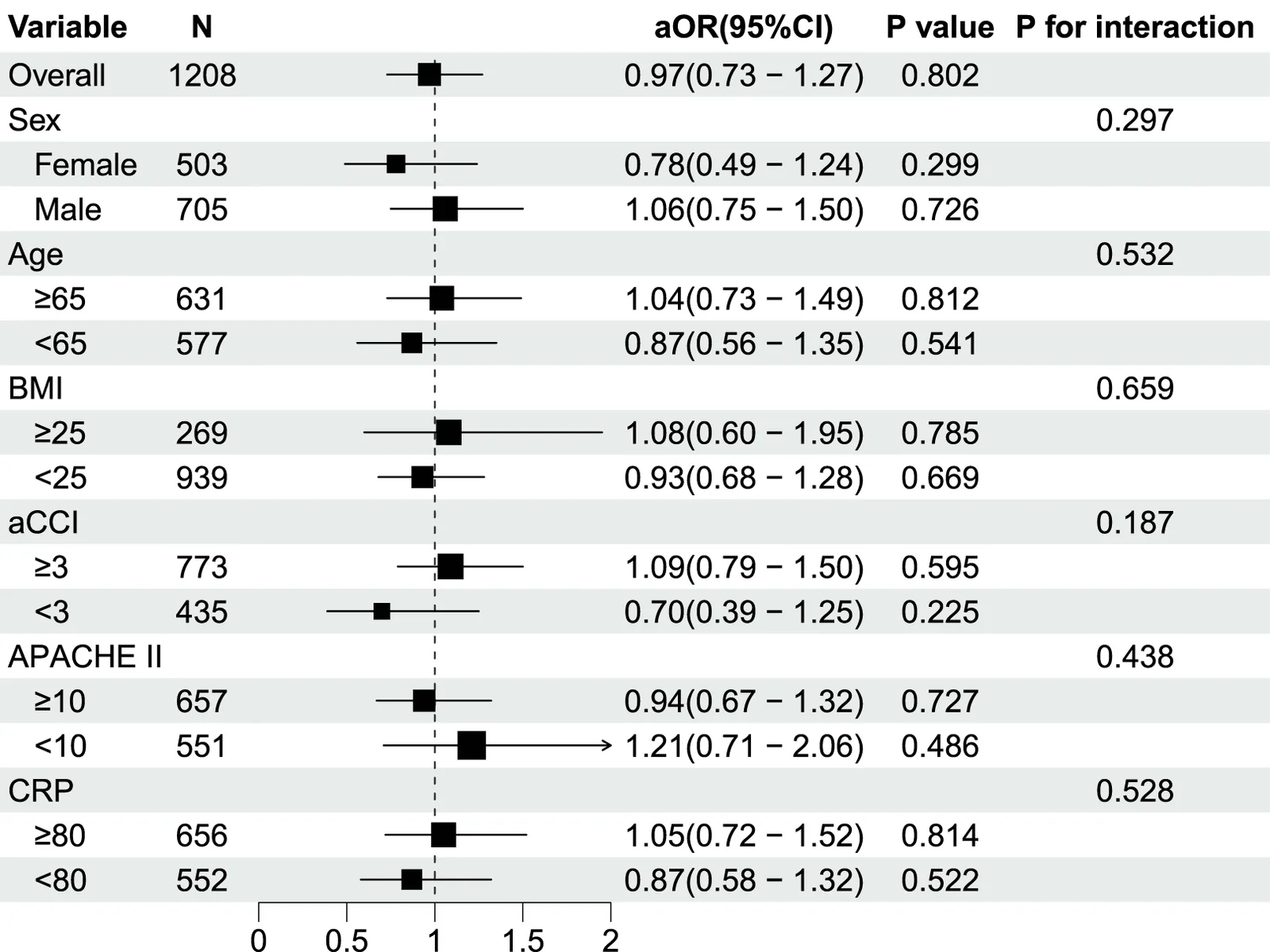
Variation in the adjusted association of overnight stay in the ED and mortality patients with sepsis according to patient and clinical factors.

#### Adherence to sepsis care bundles

To explore potential explanations for the null association between ED boarding and clinical outcomes, adherence to the 1-h sepsis bundle was analyzed. Overall compliance rates were comparable between groups: serum lactate measurement (58.7% *vs*. 52.6%; RD, −6.09 [−11.65 to −0.48]), blood cultures drawn (56.1% *vs*. 54.6%; RD, −1.47 [−7.06 to 4.14]), and antibiotic administration within 1 h (53.5% *vs*. 59.8%; RD, 6.41 [0.81 to 11.95]) (Table 3). All patients with shock received protocolized fluid resuscitation, and vasopressors were initiated immediately if target mean arterial pressure was unmet, with no between-group differences in resuscitation metrics. Overall compliance with the SSC 1-h sepsis bundle was similar in both groups (31.6% *vs*. 29.1%; RD, −2.46 [−7.63 to 2.73]) (Table 3).

**Table 3:** Comparison of compliance with each component of the 1-h bundle, according to overnight stay in the ED.

Bundle element	Total, *n* (%)	Non-overnight stay, *n* (%)	Overnight stay, *n* (%)	Difference (95% CI)
Serum lactate measurement	673 (55.7)	307 (52.6)	366 (58.7)	−6.09 [−11.65 to −0.48]
Blood cultures drawn	669 (55.4)	319 (54.6)	350 (56.1)	−1.47 [−7.06 to 4.14]
Antibiotic administration	684 (56.6)	350 (59.8)	334 (53.5)	6.41 [0.81 to −11.95]
Fluids administration in patients with hypotension	75 (100)	44 (100)	31 (100)	NA
Administration of vasopressors when indicated	66 (100)	35 (100)	31 (100)	NA
Compliance with 1-h bundle	367 (30.4)	170 (29.1)	197 (31.6)	−2.46 [−7.63 to 2.73]

## Discussion

In this retrospective cohort study of patients with sepsis, we assessed the relationship between overnight stays in the ED and clinical outcomes. In contrast to previous studies that have emphasized the risk of prolonged ED stays, our findings suggest that there were no significant differences in mortality, septic shock or length of stay between patients who spent the night in the ED and those who were admitted immediately to a ward/intensive care unit. These results persisted after rigorous adjustment for disease severity and protocol adherence, suggesting that standardized sepsis care may help mitigate risks traditionally associated with overnight stays in the ED in this observational cohort.

Previous studies have consistently associated ED overcrowding with delayed sepsis interventions and worse prognosis (Gaieski et al., 2017; Peltan et al., 2019; Shin et al., 2013). For example, delayed antibiotic administration (≥1 h) has been associated with a 9–16% increase in hourly mortality in patients with sepsis (Liu et al., 2017; Morneau et al., 2017; Ballester et al., 2018). However, adherence to the SSC 1-h bundle was comparable between groups in our cohort, which may be the reason for the absence of an association between boarding status and prognosis (Sterling et al., 2015). Although prolonged ED boarding may increase the risk of death through a multifactorial mechanism, strict adherence to the sepsis care bundle appears to counteract these adverse effects. Evidence suggests that protocol-driven strategies (*e.g*., mandatory screening and nurse-initiated lactate testing) may be effective in mitigating delays caused by ED overcrowding (Liu et al., 2017; Ballester et al., 2018; Sterling et al., 2015). In addition, environmental stressors (*e.g*., noise, limited nursing ratios) may have been offset by effective resource allocation—such as dedicated sepsis teams or rapid response protocols—ensuring continuity of critical care even during prolonged ED stays (Mohr et al., 2020; Zhou et al., 2023; Almeida et al., 2013).

The studied EDs prioritized sepsis-specific interventions regardless of boarding status. Notably, all patients with septic shock received protocolized fluid resuscitation, and vasopressors were initiated immediately if hemodynamic targets were unmet. This aligns with the understanding that early antibiotic administration and timely fluid resuscitation confer the greatest survival benefit, as they are the two most potent modifiable factors in sepsis mortality (Evans et al., 2021; Kumar et al., 2006). ProCESS (Yealy et al., 2014) and ARISE (Peake et al., 2014) landmark trials demonstrated that when these interventions were achieved within 3 h, protocolized care provided no incremental advantage over usual care. Critically, our findings suggest that rigorous compliance with sepsis care bundles may be associated with neutralization of the the excess mortality risk attributable to ED overnight stay, particularly when time-sensitive interventions are prioritized during overnight transitions of care (Liu et al., 2017; Levy, Evans & Rhodes, 2018). SSC bundle compliance has been shown to paradoxically improved during night shifts compared to daytime hours (Almeida et al., 2013). Enhanced protocol adherence during off-hours may stem from reduced ED crowding and optimized nurse-to-patient ratios, enabling more focused implementation of time-critical interventions (Zhou et al., 2023; Almeida et al., 2013). Furthermore, balanced baseline severity (APACHE II, SOFA) and comorbidities between groups reduced confounding by illness acuity, strengthening the validity of our conclusions.

Our findings have several limitations. First, as a retrospective observational study, our results can only demonstrate associations and cannot establish causality. Residual confounding is inevitable despite multivariable adjustment and propensity score weighting. Potential unmeasured confounders include nighttime ED staffing levels (*e.g*., nurse-to-patient ratio), ED crowding metrics, subtle differences in the exact timing of sepsis interventions (time to antibiotics and time to fluids), and illness severity or hemodynamic stability specifically at the time of ED presentation, even though APACHE II and SOFA scores were similar between groups (Table 1) and were adjusted for in the multivariable models. If the overnight group inadvertently included milder or more stable patients at presentation, this could have masked a potential adverse effect of overnight ED boarding. Second, while propensity score adjustment mitigated observed confounders, residual bias from unaccounted factors such as nuanced differences in antimicrobial stewardship or source control prioritization cannot be fully excluded. Third, the binary definition of “overnight ED stay” based on a midnight–8:00 AM cutoff is somewhat arbitrary. This definition may not fully reflect actual boarding duration, ED crowding intensity, or the precise timing of sepsis interventions. However, sensitivity analyses using continuous ED length of stay and overnight LOS as exposure variables (Table 2) yielded consistent null findings, supporting the robustness of our primary results. Fourth, several factors may limit the generalizability of our findings. This study reported a high rate of overnight emergency department boarding (51.7%), which may be attributed to the fact that the research was primarily conducted during the pandemic period and the emergency center of this institution had long been operating beyond capacity. These specific conditions, including pandemic-related overcrowding and high boarding prevalence, may limit the generalizability of the data to other healthcare settings or non-pandemic periods. Readers should therefore interpret our results with appropriate caution to avoid overinterpreting the findings beyond similar high-pressure emergency environments. Fifth, patients who remained in the ED overnight may have differed systematically from those admitted before midnight. They might have been clinically more stable, less likely to require immediate ICU admission, or triaged differently by clinicians. Such selection bias could have biased our results toward finding no difference between groups. Sixth, the exclusion of patients who were admitted to the wards/ICUs between 00:00–08:00 may have introduced selection bias. Finally, the multiple comparisons across outcomes and subgroups increase the risk of false-positive findings, and results should be interpreted as exploratory.

These findings suggest that, in settings with robust sepsis protocols, overnight ED boarding was not associated with worse clinical outcomes when time-sensitive interventions were consistently delivered (Evans et al., 2021). However, given the retrospective observational design of the study, causal inferences cannot be drawn. However, this should not overshadow the systemic risks of ED overcrowding, which strain resources and increase clinician burnout. Hospitals should prioritize protocol adherence during boarding periods—for example, through automated sepsis alerts or dedicated ED sepsis pathways—to standardize care (You et al., 2022). Future research should investigate whether specific subgroups, such as patients with septic shock or immunocompromising conditions, remain vulnerable to ED overnight stay effects. Prospective studies incorporating real-time monitoring of environmental stressors (*e.g*., sleep disruption, mobility restrictions) and adherence metrics could further elucidate mechanisms linking ED overnight stay to outcomes (Prendiville et al., 2020).

## Conclusion

In this retrospective cohort of sepsis patients, overnight ED boarding was not associated with increased in-hospital mortality or morbidity when care adhered to Surviving Sepsis Campaign guidelines. These results suggest that high compliance with the SSC 1-h sepsis bundle may help mitigate the potential risks of overnight ED boarding. While ED overcrowding remains a major systemic challenge, our findings indicate that, in settings with robust protocol adherence, sepsis patients may not experience worse outcomes even if they spend the first night in the ED.

## Supplemental Information

10.7717/peerj.21880/supp-1 Supplemental Information 1 Comparison of standardized mean differences for the unweighted sample, after IPTW.

10.7717/peerj.21880/supp-2 Supplemental Information 2 Variation in the adjusted association of overnight stay in the ED and mortality patients with sepsis according to patient and clinical factors.

10.7717/peerj.21880/supp-3 Supplemental Information 3 Variation in the adjusted association of overnight stay in the ED and adverse outcome patients with sepsis according to patient and clinical factors.

10.7717/peerj.21880/supp-4 Supplemental Information 4 Adjusted Association of Overnight Stay in the ED, ED LOS, ED Overnight LOS and Adverse Outcome Patients with Sepsis.

10.7717/peerj.21880/supp-5 Supplemental Information 5 APACHE II-Adjusted Sensitivity Analyses for the Association of Overnight Stay in the ED and Adverse Outcomes Among Patients with Sepsis.

10.7717/peerj.21880/supp-6 Supplemental Information 6 SOFA-Adjusted Sensitivity Analyses for the Association of Overnight Stay in the ED and Adverse Outcomes Among Patients with Sepsis.

10.7717/peerj.21880/supp-7 Supplemental Information 7 Data Collection and Sensitivity Analyses.

10.7717/peerj.21880/supp-8 Supplemental Information 8 Raw data.

## Additional Information and Declarations

### Data Availability

The following information was supplied regarding data availability:

The raw measurements are available in the Supplemental Files.
