# Supplementary material for: Overnight stay in the emergency department and mortality in sepsis patients
Source: PeerJ. 2026 Nov 10;14:e21880. doi: 10.7717/peerj.21880 (PMC13762320; doi:10.7717/peerj.21880)
Supplement: Supplementary file 1 [file peerj-14-21880-s001.pdf]

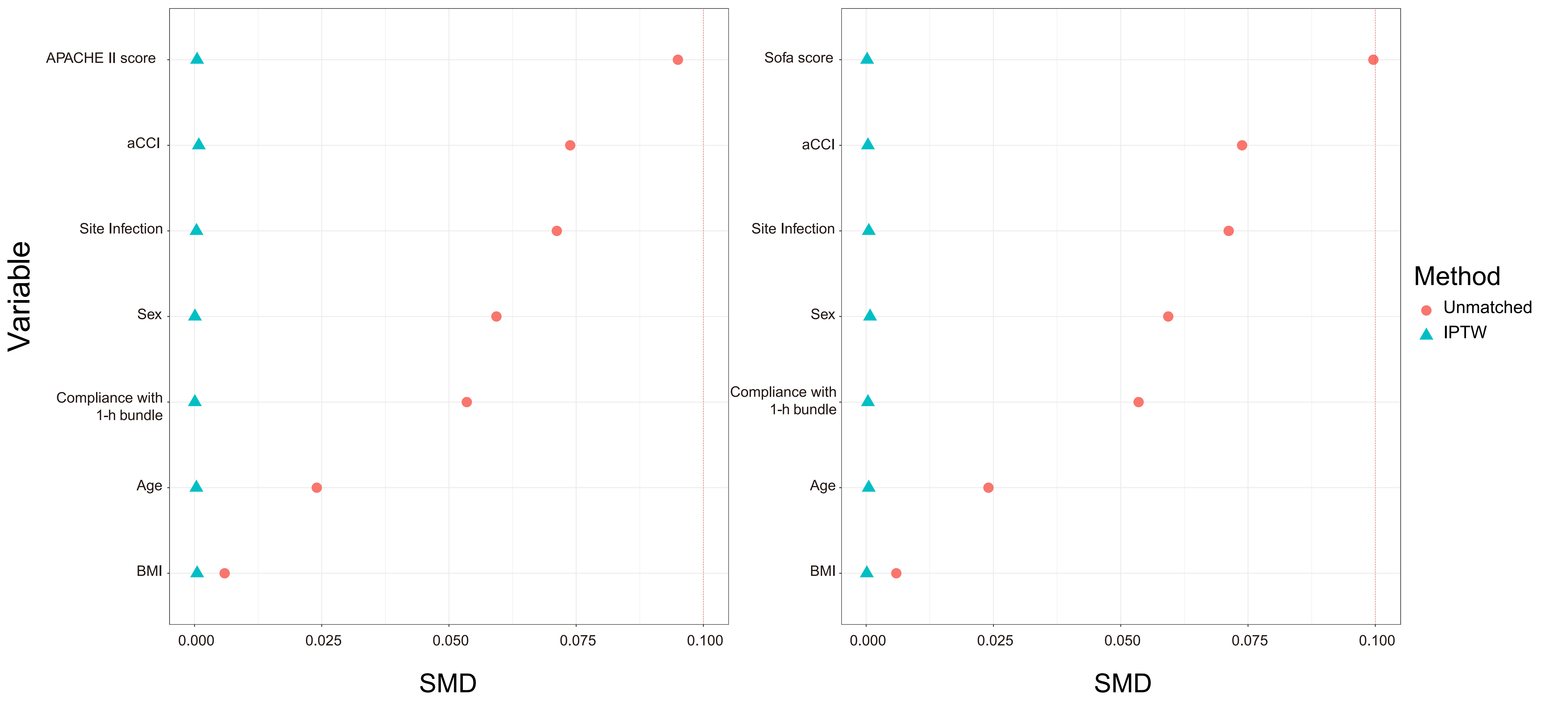

Variable
APACHE II score
aCCI
Site Infection
Sex
Compliance with 1-h bundle
Age
BMI
0.000
0.025
0.050
0.075
0.100
SMD
Sofa score
aCCI
Site Infection
Sex
Compliance with 1-h bundle
Age
BMI
0.000
0.025
0.050
0.075
0.100
SMD
Method
Unmatched
IPTW
