# Supplementary material for: Overnight stay in the emergency department and mortality in sepsis patients
Source: PeerJ. 2026 Nov 10;14:e21880. doi: 10.7717/peerj.21880 (PMC13762320; doi:10.7717/peerj.21880)
Supplement: Supplementary file 2 [file peerj-14-21880-s002.pdf]

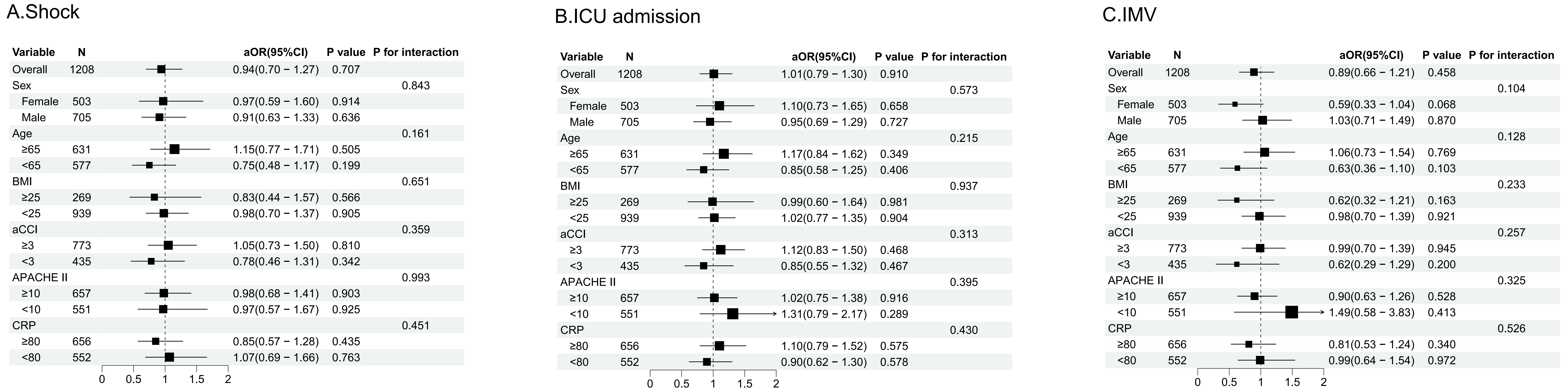
A.Shock
Variable N aOR(95%CI) P value P for interaction
Overall 1208 0.94(0.70 − 1.27) 0.707
Sex 0.843
Female 503 0.97(0.59 − 1.60) 0.914
Male 705 0.91(0.63 − 1.33) 0.636
Age 0.161
≥65 631 1.15(0.77 − 1.71) 0.505
<65 577 0.75(0.48 − 1.17) 0.199
BMI 0.651
≥25 269 0.83(0.44 − 1.57) 0.566
<25 939 0.98(0.70 − 1.37) 0.905
aCCI 0.359
≥3 773 1.05(0.73 − 1.50) 0.810
<3 435 0.78(0.46 − 1.31) 0.342
APACHE II 0.993
≥10 657 0.98(0.68 − 1.41) 0.903
<10 551 0.97(0.57 − 1.67) 0.925
CRP 0.451
≥80 656 0.85(0.57 − 1.28) 0.435
<80 552 1.07(0.69 − 1.66) 0.763
0 0.5 1 1.5 2
B.ICU admission
Variable N aOR(95%CI) P value P for interaction
Overall 1208 1.01(0.79 − 1.30) 0.910
Sex 0.573
Female 503 1.10(0.73 − 1.65) 0.658
Male 705 0.95(0.69 − 1.29) 0.727
Age 0.215
≥65 631 1.17(0.84 − 1.62) 0.349
<65 577 0.85(0.58 − 1.25) 0.406
BMI 0.937
≥25 269 0.99(0.60 − 1.64) 0.981
<25 939 1.02(0.77 − 1.35) 0.904
aCCI 0.313
≥3 773 1.12(0.83 − 1.50) 0.468
<3 435 0.85(0.55 − 1.32) 0.467
APACHE II 0.395
≥10 657 1.02(0.75 − 1.38) 0.916
<10 551 1.31(0.79 − 2.17) 0.289
CRP 0.430
≥80 656 1.10(0.79 − 1.52) 0.575
<80 552 0.90(0.62 − 1.30) 0.578
0 0.5 1 1.5 2
C.IMV
Variable N aOR(95%CI) P value P for interaction
Overall 1208 0.89(0.66 − 1.21) 0.458
Sex 0.104
Female 503 0.59(0.33 − 1.04) 0.068
Male 705 1.03(0.71 − 1.49) 0.870
Age 0.128
≥65 631 1.06(0.73 − 1.54) 0.769
<65 577 0.63(0.36 − 1.10) 0.103
BMI 0.233
≥25 269 0.62(0.32 − 1.21) 0.163
<25 939 0.98(0.70 − 1.39) 0.921
aCCI 0.257
≥3 773 0.99(0.70 − 1.39) 0.945
<3 435 0.62(0.29 − 1.29) 0.200
APACHE II 0.325
≥10 657 0.90(0.63 − 1.26) 0.528
<10 551 1.49(0.58 − 3.83) 0.413
CRP 0.526
≥80 656 0.81(0.53 − 1.24) 0.340
<80 552 0.99(0.64 − 1.54) 0.972
0 0.5 1 1.5 2

2) 0.467
