# Supplementary material for: Overnight stay in the emergency department and mortality in sepsis patients
Source: PeerJ. 2026 Nov 10;14:e21880. doi: 10.7717/peerj.21880 (PMC13762320; doi:10.7717/peerj.21880)

A.Mortality

Variable	N	aOR(95%CI)	P value	P for interaction
Overall	1208	0.97(0.73 − 1.27)	0.802	
Sex				0.297
Female	503	0.78(0.49 − 1.24)	0.299	
Male	705	1.06(0.75 − 1.50)	0.726	
Age				0.532
≥65	631	1.04(0.73 − 1.49)	0.812	
<65	577	0.87(0.56 − 1.35)	0.541	
BMI				0.659
≥25	269	1.08(0.60 − 1.95)	0.785	
<25	939	0.93(0.68 − 1.28)	0.669	
aCCI				0.187
≥3	773	1.09(0.79 − 1.50)	0.595	
<3	435	0.70(0.39 − 1.25)	0.225	
Sofa				0.657
≥3	663	0.96(0.69 − 1.34)	0.830	
<3	545	1.12(0.63 − 1.99)	0.698	
CRP				0.528
≥80	656	1.05(0.72 − 1.52)	0.814	
<80	552	0.87(0.58 − 1.32)	0.522	

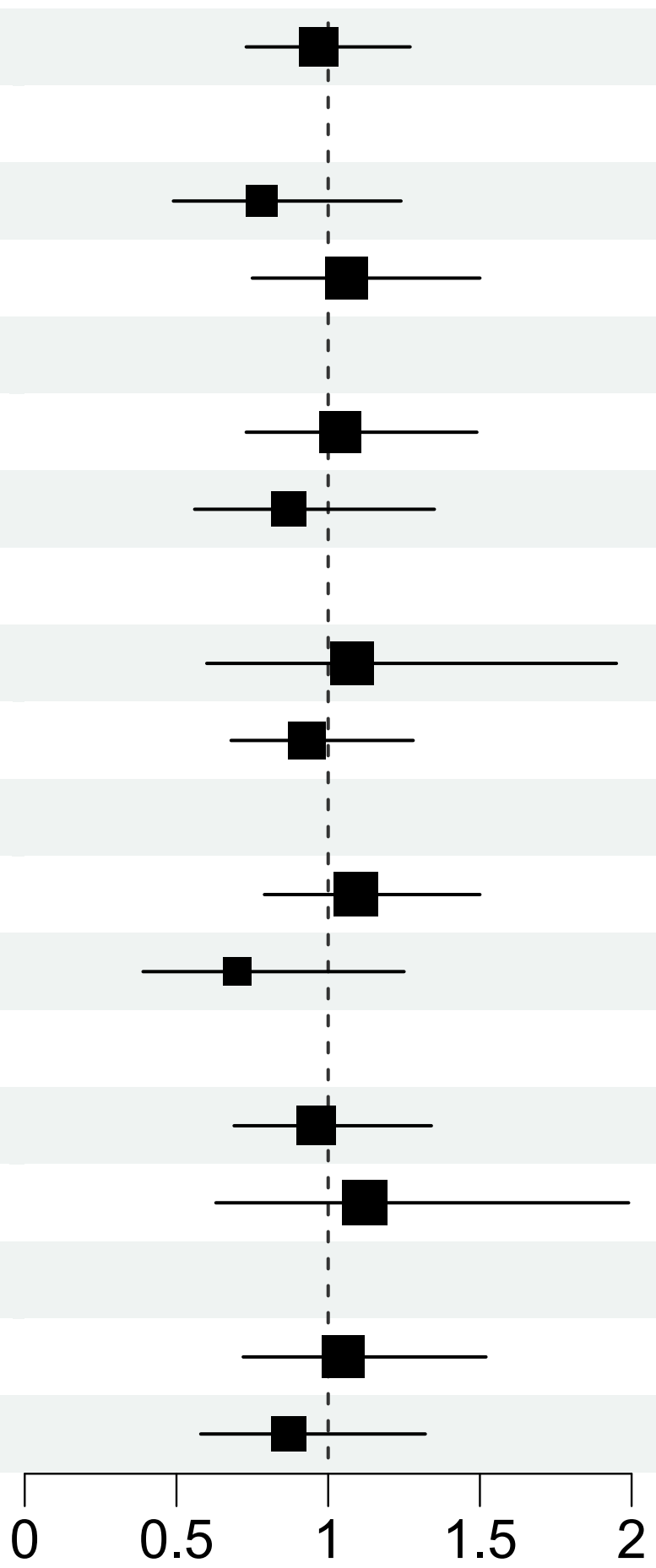

B.Shock

Variable	N	aOR(95%CI)	P value	P for interaction
Overall	1208	0.94(0.70 − 1.27)	0.707	
Sex				0.843
Female	503	0.97(0.59 − 1.60)	0.914	
Male	705	0.91(0.63 − 1.33)	0.636	
Age				0.161
≥65	631	1.15(0.77 − 1.71)	0.505	
<65	577	0.75(0.48 − 1.17)	0.199	
BMI				0.651
≥25	269	0.83(0.44 − 1.57)	0.566	
<25	939	0.98(0.70 − 1.37)	0.905	
aCCI				0.359
≥3	773	1.05(0.73 − 1.50)	0.810	
<3	435	0.78(0.46 − 1.31)	0.342	
Sofa				0.501
≥3	663	1.04(0.71 − 1.50)	0.854	
<3	545	0.83(0.50 − 1.38)	0.484	
CRP				0.451
≥80	656	0.85(0.57 − 1.28)	0.435	
<80	552	1.07(0.69 − 1.66)	0.763	

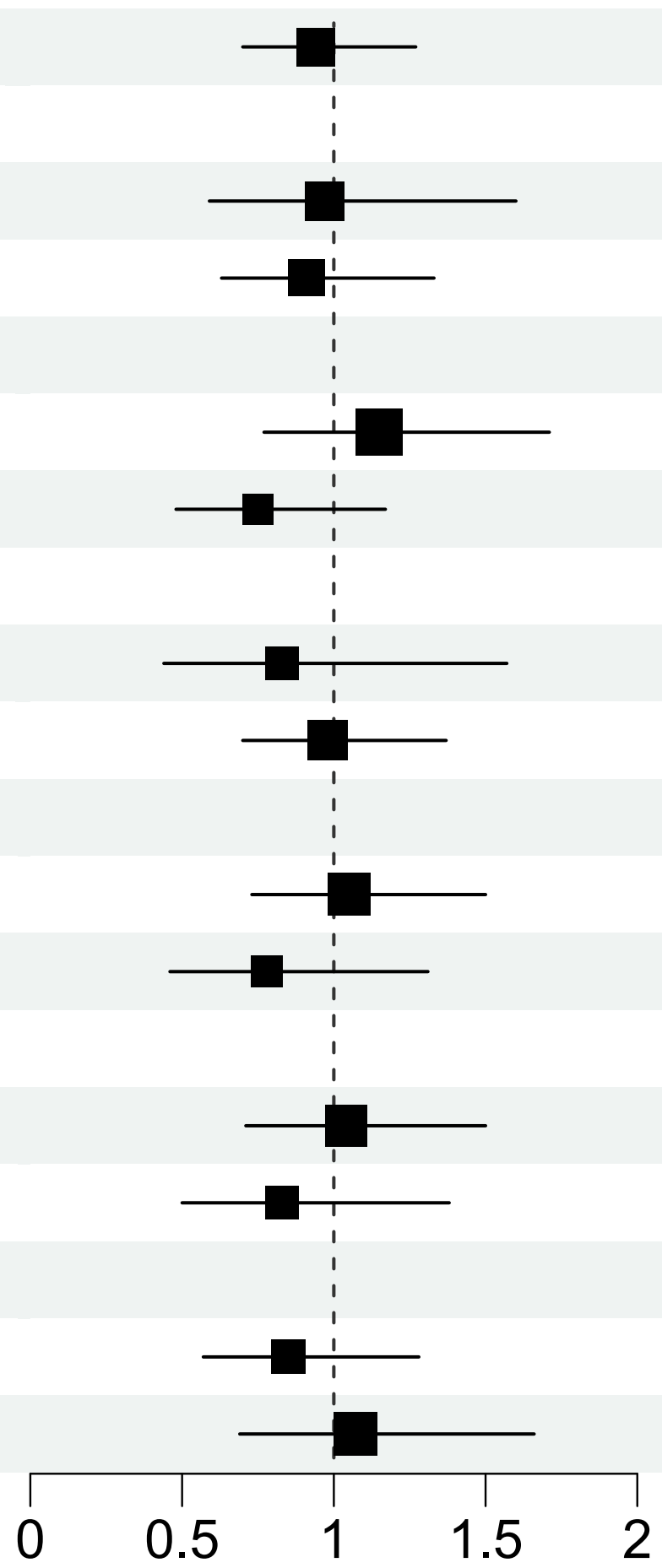

C.ICU admission

Variable	N	aOR(95%CI)	P value	P for interaction
Overall	1208	1.01(0.79 − 1.30)	0.910	
Sex				0.573
Female	503	1.10(0.73 − 1.65)	0.658	
Male	705	0.95(0.69 − 1.29)	0.727	
Age				0.215
≥65	631	1.17(0.84 − 1.62)	0.349	
<65	577	0.85(0.58 − 1.25)	0.406	
BMI				0.937
≥25	269	0.99(0.60 − 1.64)	0.981	
<25	939	1.02(0.77 − 1.35)	0.904	
aCCI				0.313
≥3	773	1.12(0.83 − 1.50)	0.468	
<3	435	0.85(0.55 − 1.32)	0.467	
Sofa				0.992
≥3	663	1.05(0.77 − 1.43)	0.755	
<3	545	1.05(0.67 − 1.63)	0.836	
CRP				0.430
≥80	656	1.10(0.79 − 1.52)	0.575	
<80	552	0.90(0.62 − 1.30)	0.578	

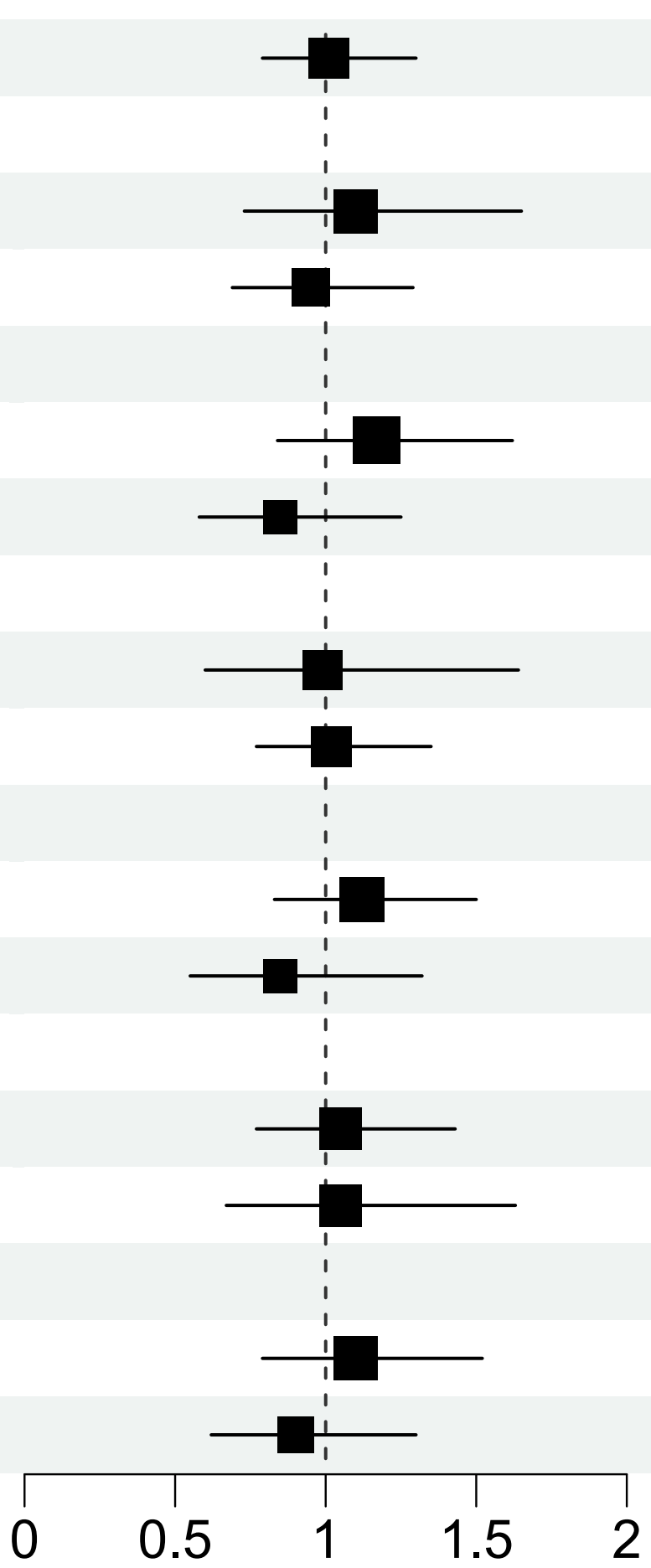

D.IMV

Variable	N	aOR(95%CI)	P value	P for interaction
Overall	1208	0.89(0.66 − 1.21)	0.458	
Sex				0.104
Female	503	0.59(0.33 − 1.04)	0.068	
Male	705	1.03(0.71 − 1.49)	0.870	
Age				0.128
≥65	631	1.06(0.73 − 1.54)	0.769	
<65	577	0.63(0.36 − 1.10)	0.103	
BMI				0.233
≥25	269	0.62(0.32 − 1.21)	0.163	
<25	939	0.98(0.70 − 1.39)	0.921	
aCCI				0.257
≥3	773	0.99(0.70 − 1.39)	0.945	
<3	435	0.62(0.29 − 1.29)	0.200	
Sofa				0.254
≥3	663	1.01(0.71 − 1.45)	0.943	
<3	545	0.65(0.33 − 1.28)	0.211	
CRP				0.526
≥80	656	0.81(0.53 − 1.24)	0.340	
<80	552	0.99(0.64 − 1.54)	0.972	

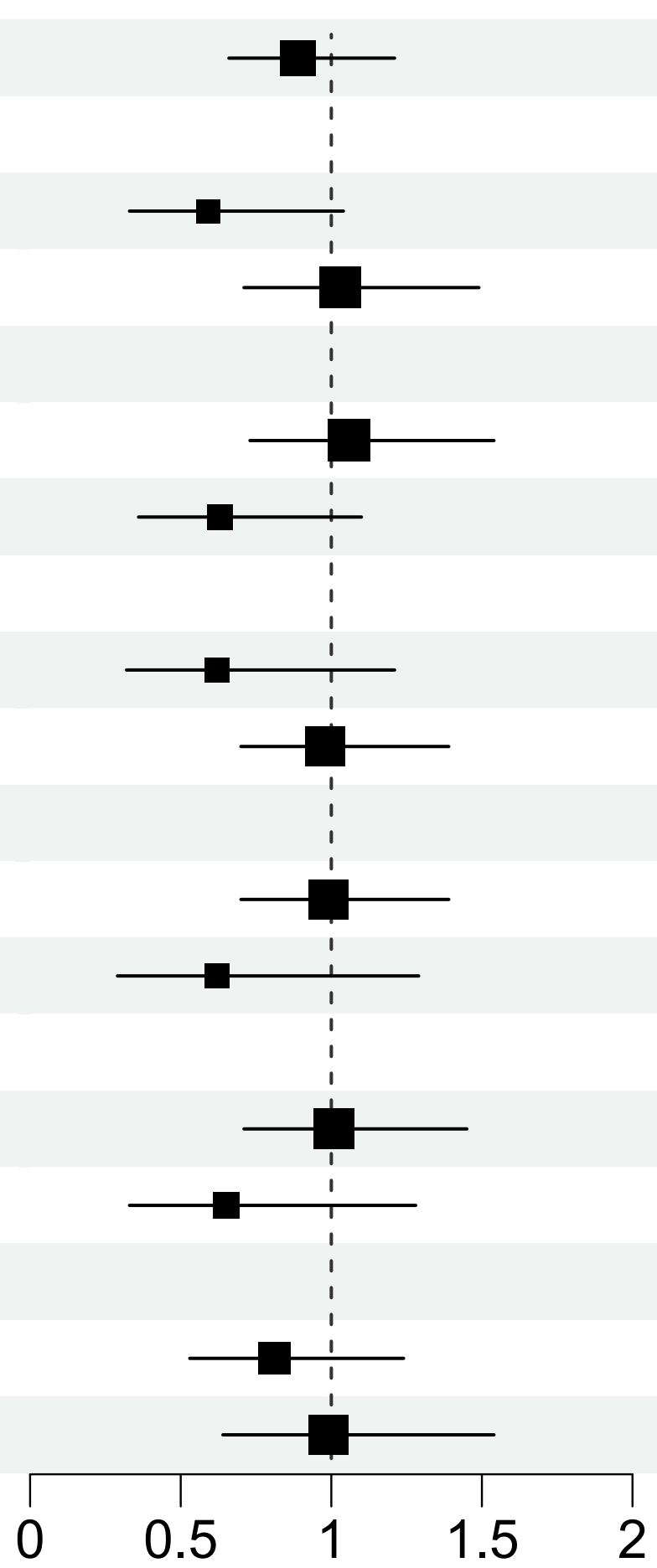

Supplement: Supplementary file 3 [file peerj-14-21880-s003.pdf]
