# Supplementary material for: Overnight stay in the emergency department and mortality in sepsis patients
Source: PeerJ. 2026 Nov 10;14:e21880. doi: 10.7717/peerj.21880 (PMC13762320; doi:10.7717/peerj.21880)
Supplement: Supplementary file 4 [file peerj-14-21880-s004.docx]

Supplementary Table 1 Adjusted Association of Overnight Stay in the ED, ED LOS, ED Overnight LOS and Adverse Outcome Patients with Sepsis.

Adjusted Variables: Age, Sex, BMI, aCCI, SOFA score, Site of Infection, Compliance with 1-h bundle.

| Variable | Mortality | | Shock | | ICU admission | | IMV | | ^*^ ICU LOS | | Hospital LOS | |
| --- | --- | --- | --- | --- | --- | --- | --- | --- | --- | --- | --- | --- |
|  | Adjusted  OR (95%CI) | *P* Value | Adjusted  OR (95%CI) | *P* Value | Adjusted  OR (95%CI) | *P* Value | Adjusted  OR (95%CI) | *P* Value | β Coefficient  (95% CI) | *P* Value | β Coefficient  (95% CI) | *P* Value |
| Overnight Stay in the ED | 1.089 (0.804-1.479) | 0.582 | 1 .000(0.736-1.361) | 0.998 | 1.15 0(0.873-1.517) | 0.322 | 0.994 (0.699-1.414) | 0.973 | -4.818(-15.476-5.839) | 0.375 | -0.978(-4.588-2.631) | 0.595 |
| ED LOS | 1.007 (0.994-1.019) | 0.299 | 0.998 (0.985-1.011) | 0.801 | 1.006 (0.995-1.018) | 0.287 | 1.004 (0.989-1.019) | 0.576 | -0.095(-0.555-0.364) | 0.684 | -0.035(-0.188-0.119) | 0.658 |
| ED Overnight LOS | 1.011 (0.981-1.043) | 0.463 | 0.992 (0.961-1.023) | 0.609 | 1.004 (0.976-1.032) | 0.769 | 0.996 (0.960-1.033) | 0.834 | -0.343(-1.429-0.744) | 0.536 | -0.129(-0.495-0.238) | 0.490 |

^*^ This study was confined to the analysis of patients admitted to the ICU.
