# Supplementary material for: Overnight stay in the emergency department and mortality in sepsis patients
Source: PeerJ. 2026 Nov 10;14:e21880. doi: 10.7717/peerj.21880 (PMC13762320; doi:10.7717/peerj.21880)
Supplement: Supplementary file 5 [file peerj-14-21880-s005.docx]

Supplementary Table 2 Sensitivity Analyses of the Adjusted Association of Overnight Stay in the ED and Adverse Outcome Patients with Sepsis.

Adjusted Variables: Age, Sex, BMI, aCCI, APACHE II score, Site of Infection, Compliance with 1-h bundle.

| Variable | Mortality | | Shock | | ICU admission | | IMV | | ^*^ICU LOS | | Hospital LOS | |
| --- | --- | --- | --- | --- | --- | --- | --- | --- | --- | --- | --- | --- |
|  | Adjusted  OR (95%CI) | *P* Value | Adjusted  OR (95%CI) | *P* Value | Adjusted  OR (95%CI) | *P* Value | Adjusted  OR (95%CI) | *P* Value | β Coefficient  (95% CI) | *P* Value | β Coefficient  (95% CI) | *P* Value |
| Overnight Stay in the ED | 1.092(0.807-1.479) | 0.571 | 1.022(0.749-1.395) | 0.892 | 1.361(0.983-1.891) | 0.064 | 1.047(0.708-1.549) | 0.818 | -4.348(-15.203-6.507) | 0.431 | -0.644(-4.283-2.995) | 0.729 |

^*^ This study was confined to the analysis of patients admitted to the ICU.
