# Supplementary material for: Overnight stay in the emergency department and mortality in sepsis patients
Source: PeerJ. 2026 Nov 10;14:e21880. doi: 10.7717/peerj.21880 (PMC13762320; doi:10.7717/peerj.21880)
Supplement: Supplementary file 6 [file peerj-14-21880-s006.docx]

Supplementary Table 3 Sensitivity Analyses of the Adjusted Association of Overnight Stay in the ED and Adverse Outcome Patients with Sepsis.

Adjusted Variables: Age, Sex, BMI, aCCI, SOFA score, Site of Infection, Compliance with 1-h bundle.

| Variable | Mortality | | Shock | | ICU admission | | IMV | | ICU LOS | | Hospital LOS | |
| --- | --- | --- | --- | --- | --- | --- | --- | --- | --- | --- | --- | --- |
|  | Adjusted  OR (95%CI) | *P* Value | Adjusted  OR (95%CI) | *P* Value | Adjusted  OR (95%CI) | *P* Value | Adjusted  OR (95%CI) | *P* Value | β Coefficient  (95% CI) | *P* Value | β Coefficient  (95% CI) | *P* Value |
| Overnight Stay in the ED | 1.092 (0.805-1.482) | 0.572 | 1.001 (0.737-1.361) | 0.993 | 1.149 (0.872-1.514) | 0.324 | 0.991 (0.697-1.409) | 0.960 | -4.836(-15.798-6.126) | 0.386 | -0.982(-4.677-2.713) | 0.602 |
