# Supplementary material for: Overnight stay in the emergency department and mortality in sepsis patients
Source: PeerJ. 2026 Nov 10;14:e21880. doi: 10.7717/peerj.21880 (PMC13762320; doi:10.7717/peerj.21880)
Supplement: Supplementary file 7 [file peerj-14-21880-s007.docx]

**Supplementary Material on Data Collection**

The research data for this study were obtained through the following standardized procedure: First, the principal investigator of this study submitted a formal data acquisition request. Subsequently, the Hospital Information Department extracted the raw datasets from the institutional electronic medical record system. To ensure data accuracy and consistency, two qualified clinical physicians who had received specialized training in data collection protocols independently collected and cross-verified the same dataset. In instances where discrepancies were identified between the two independently collected datasets, the principal investigator conducted a comprehensive review of the conflicting entries and served as the final arbitrator to resolve discrepancies and determine the definitive values for inclusion in the analysis.

Operational Definitions of Key Variables

To ensure clarity and reproducibility, the following operational definitions were applied to study variables with potential ambiguity:

1.Overnight Stay in the ED

A patient was classified as having an overnight ED in the stay if they: (a) Were admitted to the ED before 24:00 (midnight), and (b) Were transferred to an inpatient hospital ward after 08:00 the following morning.

Exclusion criterion: Patients transferred between 00:00 and 08:00 were excluded from this classification.

2. ED LOS

Defined as the total time duration (hours) from ED registration to transfer to an inpatient ward.

3. ED Overnight LOS

Specifically quantified the cumulative time spent in the ED between 00:00 and 08:00.

**Illustrative Example**

For a patient admitted at 03:00 and transferred at 09:00:

Classification: Non-Overnight Stay in the ED (transfer occurred after 08:00 but initial admission was after 00:00)

ED LOS: 6 hours (03:00–09:00)

ED Overnight LOS: 5 hours (03:00–08:00)

**Supplementary Material on Sensitivity Analyses**

To address potential confounding by indication in evaluating the association between overnight stay in the ED and adverse outcomes in sepsis patients, we performed sensitivity analyses using inverse probability of treatment weighting (IPTW). The propensity score was estimated through multivariable logistic regression modeling the probability of Overnight Stay in the ED (exposure group: ED admission before 24:00 with ward transfer after 08:00) versus Non-Overnight Stay in the ED (control group: transferred before 08:00). Covariates included in the propensity score model were identical to those adjusted for in the primary multivariable regression analysis. After IPTW, all covariates exhibited standardized mean difference (SMD) below the threshold of 0.1, indicating well-balanced variables between the groups. (Supplementary Figure 1). The final results were consistent with the results of our main study (Supplementary Table 2 and Supplementary Table 3).
